# *Ixodes pacificus* Ticks Maintain Embryogenesis and Egg Hatching after Antibiotic Treatment of *Rickettsia* Endosymbiont

**DOI:** 10.1371/journal.pone.0104815

**Published:** 2014-08-08

**Authors:** Andre H. Kurlovs, Jinze Li, Du Cheng, Jianmin Zhong

**Affiliations:** Department of Biological Sciences, Humboldt State University, Arcata, California, United States of America; University of Minnesota, United States of America

## Abstract

*Rickettsia* is a genus of intracellular bacteria that causes a variety of diseases in humans and other mammals and associates with a diverse group of arthropods. Although *Rickettsia* appears to be common in ticks, most *Rickettsia*-tick relationships remain generally uncharacterized. The most intimate of these associations is *Rickettsia* species phylotype G021, a maternally and transstadially transmitted endosymbiont that resides in 100% of *I. pacificus* in California. We investigated the effects of this *Rickettsia* phylotype on *I. pacificus* reproductive fitness using selective antibiotic treatment. Ciprofloxacin was 10-fold more effective than tetracycline in eliminating *Rickettsia* from *I. pacificus*, and quantitative PCR results showed that eggs from the ciprofloxacin-treated ticks contained an average of 0.02 *Rickettsia* per egg cell as opposed to the average of 0.2 in the tetracycline-treated ticks. Ampicillin did not significantly affect the number of *Rickettsia* per tick cell in adults or eggs compared to the water-injected control ticks. We found no relationship between tick embryogenesis and rickettsial density in engorged *I. pacificus* females. Tetracycline treatment significantly delayed oviposition of *I. pacificus* ticks, but the antibiotic’s effect was unlikely related to *Rickettsia*. We also demonstrated that *Rickettsia*-free eggs could successfully develop into larvae without any significant decrease in hatching compared to eggs containing *Rickettsia*. No significant differences in the incubation period, egg hatching rate, and the number of larvae were found between any of the antibiotic-treated groups and the water-injected tick control. We concluded that *Rickettsia* species phylotype G021 does not have an apparent effect on embryogenesis, oviposition, and egg hatching of *I. pacificus*.

## Introduction

Bacterial endosymbiosis is ubiquitous in arthropods and ranges from mutualism to parasitism [1]–[3]. Bacterial symbionts may exhibit multiple effects on their hosts [3] – for example, the most well studied arthropod symbiont *Wolbachia* is a reproductive manipulator that also increases fitness of the mosquito *Aedes albopictus* [4]. The most intimate association between bacteria and arthropods is heritable obligate mutualism, where the host cannot survive and/or produce viable offspring without the symbiont [5]. A classic example of obligate symbiosis is *Buchnera*, which provides essential amino acids to the pea aphid *Acyrthosiphon pisum* [6]. However, most arthropod endosymbionts appear to be facultative, i.e. not strictly required for the host’s survival and reproduction [3].

*Rickettsia* is a genus of gram-negative obligate intracellular bacteria within the class Alphaproteobacteria. Rickettsiae live mainly inside arthropods, but associate with a diverse range of hosts, including leeches and hydra [7]. Arthropod rickettsiae are generally facultative symbionts that display a wide range of relationships with their hosts. *Rickettsia* can be mutualistic, e.g. by protecting an aphid host against a fungal pathogen [8]. *Rickettsia* can also be a reproductive manipulator – a species of *Rickettsia* causes parthenogenesis in the parasitoid wasp *Pnigalio soemius* [9]. Reproductive manipulation that increases the female:male ratio of arthropod offspring is a common strategy among bacterial endosymbionts in arthropods, as endosymbionts’ heritability in the host lineage is usually strictly maternal [1]. Though many *Rickettsia* species have no reported vertebrate pathogenicity, the genus is best known as a pathogen of humans and cattle [10]. Classification of pathogenic rickettsiae is largely coincident with the diseases the bacteria cause in humans: spotted fever group (SFG) rickettsiae are known to cause spotted fevers while the typhus group is responsible for endemic typhus [11], [12]. SFG *Rickettsia* species are primarily vectored by the ixodid (“hard”) ticks and have been found on all continents but Antarctica [13].

The effects of rickettsiae on ticks are dependent on the specific *Rickettsia* and the tick species involved, but general patterns remain unavailable since few *Rickettsia*-tick relationships have been investigated. Natural infection with *R. montanensis*, *R. bellii* and *R. rhipicephali* decreases fitness of *Dermacentor andersoni* whereas laboratory infection with *R. rickettsii* is detrimental to the tick, with 97.5% of infected progeny from *D. andersoni* not surviving to adulthood [14]. Similarly, *R. conorii* induces mortality in laboratory-infected *Rhipicephalus sanguineus* ticks [13]. Reproductive manipulation is a possibility as endosymbiotic *R. bellii* universally infects a strictly parthenogenetic tick species, *Amblyomma rotundum* [15]. However, *Rickettsia* has been implicated in increased larval motility of the ticks *Ixodes scapularis*, *Amblyomma americanum,* and *D. andersoni,* likely making it easier for the tick species to access hosts [16].

*Rickettsia* bacteria have been found in several *Ixodes* species of ixodid ticks. *Rickettsia* species were recently reported in adults and nymphs of *Ixodes ricinus* from northern Italy [17]. In *I. scapularis*, 16S rDNA metagenomics revealed that *Rickettsia* is the most prevalent (present in 77% of all adults and nymphs studied) identified bacterial genus in Texas populations of *I. scapularis,* and the second most prevalent (83%) in New York populations [18]. However, the closest known association between *Rickettsia* and *Ixodes* is a recently discovered *Rickettsia* species (phylotype G021) that is universally present and 100% maternally transmitted in California populations of *Ixodes pacificus* [19], [20]. The ubiquitous prevalence and vertical transmission of the *Rickettsia* phylotype suggest a likely mutualistic role in the tick species.

*I. pacificus* transmits several bacterial pathogens of humans, dogs and cattle in western North America, and particularly in California where it has been reported in 56 out of 58 counties [21]. *I. pacificus* has four life cycle stages: egg, larva, nymph and adult. Larvae and nymphs require a blood meal for molting, while adult females require it for oviposition. In laboratory settings, the duration of the *I. pacificus* life cycle strongly depends on temperature. The length of the *I. pacificus* preoviposition period, for example, varies from an average of 9 days at 26°C to 70 days at 12°C [22]. *Ixodes* females’ egg counts are also highly variable, ranging from several thousand eggs per female [23], to less than eight hundred [24]. *I. pacificus* eggs can have a nearly 100% hatching rate if conditions are favorable; high humidity of at least 85% is preferred as eggs tend to die from desiccation in dry habitats [22].

Removal of symbionts using antibiotic injection has been successfully used to study functions of bacterial symbioses in ticks and aphids [25], [26]. In the lone-star tick *A. americanum,* injection with tetracycline and rifampin demonstrated that the tick species’ only known heritable endosymbiont, the bacteria *Coxiella*, are mutualists that contribute to *A. americanum*’s fecundity [26]. Selective antibiotic treatment is a strategy where antibiotics target specific groups of bacteria to determine how the individual groups of microorganisms affect the host. Ampicillin injection selectively removed *Rickettsia* in the second-generation *A. pisum* offspring and showed that *Rickettsia* decreased the aphid’s fitness by lowering the density of the aphid’s obligate symbiont *Buchnera* [25].

In this study, we investigated the effect of *Rickettsia* species phylotype G021 on reproductive fitness of *I. pacificus* adult females using selective antibiotic treatments. Three antibiotics were selected for the study: tetracycline, ciprofloxacin (a fluoroquinolone antibiotic) and ampicillin. The antibiotics were chosen based on their effectiveness in treating rickettsiae and on their mode of action. Tetracycline and fluoroquinolone antibiotics have been successfully used for treating spotted fever patients [27], and *Rickettsia* species in vitro [28]. We chose two effective antibiotics for two reasons: to control for the possibility of an antibiotic affecting *I. pacificus* independently of rickettsiae, and to use antibiotics with different modes of action. Fluoroquinolones kill bacteria by interfering with DNA topoisomerase IV and DNA gyrase [29], enzymes required for double-stranded DNA synthesis. Tetracyclines are, in contrast, bacteriostatic agents that inhibit protein synthesis by binding to the bacterial 30S ribosome, particularly the tRNA-binding site [29], [30]. Ampicillin was chosen as an antibiotic control in this study. Despite the antibiotic’s ability to affect *Rickettsia* in the second-generation offspring in *A. pisum* [25], ampicillin cannot be used to treat patients infected with rickettsial diseases [31] and is ineffective in vitro on tick-borne intracellular bacteria *Ehrlichia* and *Anaplasma* [32].

We assessed the effectiveness of different antibiotic treatment on the *Rickettsia* phylotype and determined the effects of the antibiotics on egg hatching and larval counts. Our specific research questions were as follows: (1) Which antibiotic, tetracycline or ciprofloxacin, would be more effective in treating *Rickettsia* phylotype G021 in *I. pacificus*? (2) Can ampicillin be used as an antibiotic control ineffective on *Rickettsia* phylotype G021 in *I. pacificus*? (3) Does decreasing rickettsial density in the engorged tick female affect embryogenesis, i.e. prolong the period of time before an engorged female starts ovipositing and/or have an effect on the number of offspring? (4) Does the *Rickettsia* phylotype affect egg hatching of *I. pacificus*? A broader goal of our study was to gain insights into the relationship between an important arthropod disease vector and its bacterial symbiont, with potential future implications for disease control.

## Materials and Methods

### Ticks and tick feeding

Adult *I. pacificus* were collected by flagging in mixed evergreen and oak woodland near Blue Lake in Humboldt County, California (GPS coordinate: N 40 55.200 W 123 50.400). No specific permissions were required to collect ticks from the site, and no endangered, or protected species were involved in the fieldwork conducted. The ticks were collected by three people on two trips, one in late winter and one in early spring of 2011, with roughly two hours dedicated to tick collection during each trip. Ticks were identified to the species level based on morphologic characteristics under a dissecting microscope [33] until 101 male and 101 female *I. pacificus* were obtained for the study. Ticks were then kept in polystyrene containers (Thermo Fisher Scientific, Waltham, MA) inside a desiccator with ambient temperature and day:night cycles, and 95% relative humidity.

**Figure 1:**
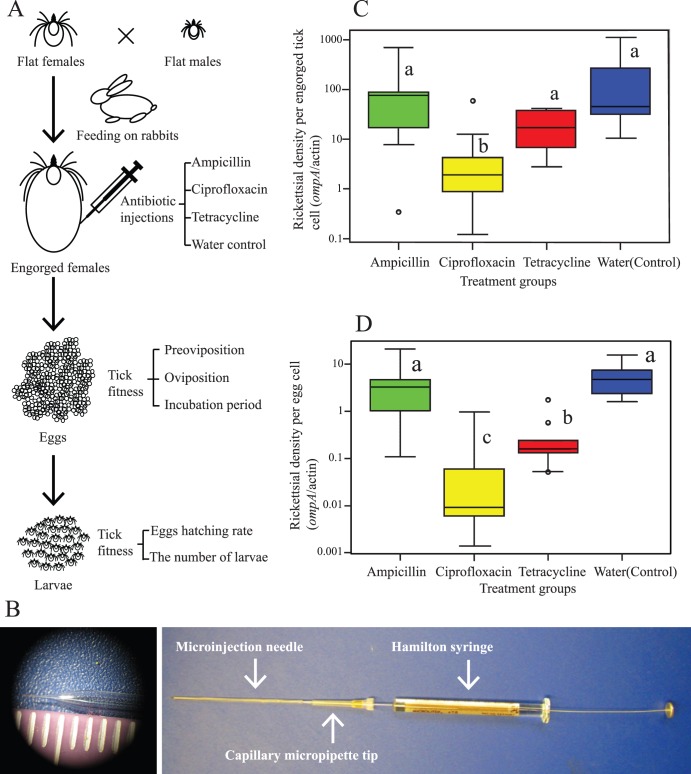
Illustration of the experimental design and the antibiotic injection results. 1A) Experimental design outline, represented as a flow chart. 1B) Tick injection apparatus. Solution is loaded into the microinjection needle using the capillary pipette tip until the needle is full. The tip is then left inside the glass needle and the syringe with a 23-gauge needle is fitted into the capillary pipette tip. 1C) and 1D) Box-whisker plots of rickettsial densities (gene copy ratios of *Rickettsia ompA* to *Ixodes pacificus actin*) detected by qPCR in (C) spent females and (D) eggs from the four treatment groups. In both (C) and (D), the same letter next to two plots informs of statistically insignificant difference between the treatment groups (*P*>0.05), meaning that lack of identical letters stipulates significance (*P*<0.05).

Adult *I. pacificus* were fed on five male New Zealand white rabbits (*Oryctolagus cuniculus* (Figure 1A). All procedures followed protocols approved by the Institutional Animal Care and Use Committee at Humboldt State University (IACUC protocol 10/11.B.45-A), and all efforts were made to minimize discomfort. Four tin cans with seamless lids (Freund Containers, Lisle, IL) were glued to shaved spots on each rabbit’s back using cyanoacrylate glue. Up to five male and five female ticks were put in each tin can. The ticks were allowed to mate on the rabbits until engorgement.

### Tick antibiotic injection and determination of hatching fitness

Upon engorgement, female ticks dropped from the rabbits on their own [34], [35] and were divided into four treatment groups of roughly equal mean weight: ampicillin, ciprofloxacin, tetracycline, and water (negative control). Engorged female ticks were injected into the hemocoel between the first and second legs [26] using microinjection needles pulled by P-97 Sutter Glass needle puller (Sutter Instrument Co, Novato, CA). To inject the ticks, we designed an injection apparatus based on commercially available components (Figure 1B). The microinjection needles were loaded with antibiotics or water using a micropipette with a disposable capillary 20 µl pipette tip (Eppendorf, Hamburg, Germany) that was eventually inserted into the needle, left in place, and filled to the top with the solution. A 10 µl Hamilton syringe (Hamilton, Reno, NV) with a 23-gauge needle was then inserted into the capillary pipette tip. All three compartments were connected to each other firmly by twisting and pushing. The syringe plunger was used to pull antibiotic solution or water into the glass and to inject the contents into ticks under a dissecting microscope (Figure 1B). Three µl of 10 mg/ml antibiotic solution (or water) was injected into ticks, and the needle was left inside tick body for 30 sec before being slowly withdrawn. Injected ticks were put back into individual polystyrene containers in the desiccator and monitored for oviposition daily. Time to oviposition or the preoviposition period was recorded as the period between tick injection and the beginning of oviposition. Ticks that died shortly after injection (likely as a result of injection stress) were excluded from the study without affecting statistical analysis, as all such deaths happened before oviposition in any of the groups commenced.

After completion of oviposition and subsequent death, each spent female, and 10 randomly selected viable eggs from each female, were preserved in 70% ethanol and stored at −20°C for further molecular analysis. The remaining eggs were left in the original polystyrene containers inside the desiccators and were monitored daily until hatching was complete. The incubation period was defined as the time from the deposition of the first egg until the completion of egg hatching into larvae. After all the larvae had hatched, the containers were placed in −20°C to freeze the larvae. The larvae and the remaining unhatched eggs were then counted under a dissecting microscope. Ticks that survived injection, but died without laying eggs were not included in the analysis to determine the difference in the incubation period, hatching rate, or the number of larvae, between treatment groups.

### DNA extraction and quantitative PCR (qPCR)

The spent *I. pacificus* females and their eggs (10 from each female) were pulverized in liquid nitrogen prior to DNA extraction. Each spent female was frozen in liquid nitrogen, and then split into four parts using a sterile scalpel; each part was treated separately for extraction purposes to improve tissue lysis and increase overall DNA yield. Each of the 10 eggs was punctured with a sterile pestle (Qiagen, Valencia, CA) before extraction. DNA was extracted using DNeasy Blood and Tissue Kit (Qiagen, Valencia, CA), as previously described [36]. One mock DNA extraction was performed for every 10 extractions from tick samples or eggs as a contamination control. Mock extractions contained distilled nuclease-free water instead of a tick or an egg sample, and were carried out according to the protocol used for other samples during DNA extraction.

To determine rickettsial prevalence and density in ticks, an outer membrane protein (*ompA*) gene of *Rickettsia* species phylotype G021 and the beta-actin biosynthetic (*actin*) gene of *I. pacificus* were qPCR-amplified using the ABI-7300 Real-Time PCR System (Life Technologies, Foster City, CA), as previously described [20]. Primer pairs and probe were designed against a divergent sequence of the single copy *ompA* gene to differentiate between phylotype G021 and the rare *Rickettsia* phylotype G022 [37]. Rickettsial density of *I. pacificus* was defined as the ratio of rickettsial *ompA* copies per *I. pacificus actin* copies. All qPCR reactions were set up as follows: 4.5 µl extracted tick DNA was mixed with 7.5 µl qPCR Master Mix Plus (AnaSpec, Fremont, CA), 0.5 µl of 5 µM primers and probe, and 1.5 µl 0.4% bovine serum albumin (BSA), yielding 15 µl total reaction volume. The reactions were run in duplicates in 96-well plate qPCR plates (Life Technologies, Foster City, CA). Four negative control reactions were run on each 96-well plate to ensure that the reagents were not contaminated with template DNA.

Standard curves for the *ompA* gene of *Rickettsia* species phylotype G021 and the *actin* gene of *I. pacificus* were constructed based on gene products cloned into Psc-A-amp/kan plasmid using topoisomerase-based StrataClone™ PCR Cloning Kit (Agilent Technologies, Santa Clara, CA). For cloning purposes, amplicons of the *ompA* gene of phylotype G021 or the *actin* gene *of I. pacificus* were each ligated into the Psc-A-amp/kan plasmid, as specified by the manufacturer’s instructions outlined in the manual for the StrataClone PCR Cloning Kit (Agilent Technologies, Santa Clara, CA). Plasmid DNA was isolated from the transformants using the Promega Wizard Plus SV Plasmid Purification System (Promega, Madison, WI) using the manufacturer’s protocol. Concentration of the plasmid DNA was determined by measuring absorbance at the wavelength of 260 nm using the Nanodrop ND-1000 Spectrophotometer (Thermo Fisher Scientific, Waltham, MA). The solution containing a known concentration of *ompA* or *actin* DNA was then 10-fold serially diluted to produce standards that were run in duplicates in each qPCR reaction to generate a standard curve. Based on the standard curve for each gene, experimental qPCR data were used to determine gene copy numbers for the *ompA* gene of phylotype G021 and the *actin* gene of *I. pacificus* in adult tick/egg DNA samples. The resultant copy numbers were used to estimate rickettsial prevalence and density of each spent female and its ten randomly selected eggs.

### Statistical analyses

R Statistical Software version 3.0.2. was used to analyze data [38]. Specifically, the Anderson-Darling test [39] was used to check if the data were normally distributed, and Levene’s test [40] was performed to test for equal variances. To normalize data, log transformations were applied to copy numbers of rickettsial densities that varied by orders of magnitude; arcsine transformations were applied to hatching rates (percentages; [41]), and the square root transformation was used for the number of larvae.

Linear regression analysis was performed to determine the relationship between egg and adult rickettsial densities. Spearman correlation analyses were used to determine correlative relationships between other variables. Kaplan-Meier analysis, a common method to analyze time-to-event data [42], was employed to compare time to oviposition and incubation periods of the four treatment groups. The Hmisc package for R was used to generate confidence intervals for the medians in the Kaplan-Meier analysis [43]. The Log-rank test with Bonferroni correction was applied to make pairwise comparisons between preoviposition periods and incubation periods of different groups. One-way analysis of variance (ANOVA) with the Tukey-Kramer pairwise comparison test was used to compare the four treatment groups with respect to rickettsial densities, hatching rate, and the number of larvae.

## Results

### Tick feeding, injection and effects of antibiotics on *Rickettsia* species phylotype G021

Out of the 101 female ticks that fed on rabbits, 89 successfully fed to engorgement. Twenty-one of the 89 engorged ticks died shortly after dropping off, leaving 68 for injection. The engorged females were then split into four groups according to their mean weight of ∼170 mg in each group. Forty-four of the 68 injected ticks laid eggs that hatched into viable larvae. The 44 ticks were distributed as follows: 12 in the ampicillin group, 14 in the ciprofloxacin group, 9 in the tetracycline and the control groups. Excluding dead ticks from statistical analyses did not create a significant difference between the mean weights of the groups (Kruskal-Wallis test, *P*>0.80). This implies that no weight discrepancy bias (where a particular group would end up with a disproportionally larger number of heavy ticks that lay more eggs) was introduced into the analysis by eliminating the ticks.

Decrease in rickettsial density as a result of antibiotic treatment was assessed by comparing the ratio of copy numbers of the rickettsial outer membrane protein gene (*ompA*) to *I. pacificus* beta-actin biosynthetic gene (*actin*) in spent females and eggs between treatment groups (*ompA/actin*). Spent females in the water control group had the highest mean (95% confidence interval (CI)) rickettsial density of 79.4 (23.4–275.4) *ompA/actin*. Spent females in the ampicillin-injected group had the second highest density with 38.0 (11.5–131.8) *ompA/actin*, followed by the tetracycline-injected group’s density of 14.5 (6.9–30.9), and the lowest density of 2.0 (0.9–4.9) in the ciprofloxacin-injected group. Spent females in the ciprofloxacin-injected females had significantly lower rickettsial density than females in any other group (ANOVA, *P*<0.0001, *df* = 43; Tukey-Kramer Test, *P*<0.05) (Figure 1C). The rickettsial densities of spent females in the rest of the groups were not significantly different from each other (Tukey-Kramer Test, *P*>0.05).

Rickettsial density in eggs was lower than in spent females (Student’s Paired t-test, *P*<0.05), with the *ompA/actin* ratio of the control group at 4.7 (2.5–8.5) being the highest. The ampicillin treated group followed with a mean density of 2.2 (0.9–5.6), higher than that of the tetracycline group at 0.2 (0.09–0.5), which was 100 times higher than the density of 0.02 (0.007–0.05) in the ciprofloxacin group. In contrast to spent females, rickettsial densities in eggs coming from adult female ticks treated with tetracycline and ciprofloxacin were significantly lower than those in either the water control or the ampicillin group (ANOVA, *P*<0.0001) (Figure 1D). In addition, eggs in the ciprofloxacin group had a significantly lower rickettsial density than eggs from the tetracycline group (Tukey-Kramer Test, *P*<0.05). Rickettsial densities in eggs demonstrated a significant linear relationship with rickettsial densities in the respective spent females (*R^2^* = 0.57, *P*<0.001).

Ciprofloxacin-injected ticks were the only group of engorged females that laid *Rickettsia*-free eggs. Cohorts of 10 randomly selected eggs from each of the 14 ovipositing ciprofloxacin-treated females were screened for *Rickettsia* species phylotype G021 by molecular analyses. Only three of the 14 egg cohorts of ciprofloxacin-injected ticks were 10 of 10 (100%) *Rickettsia*-positive (Table 1). Rickettsial prevalence in the remaining 11 egg cohorts varied from 0% to 90%. The prevalence of rickettsiae in the egg cohorts showed no relationship with the overall hatching rate of the respective ticks’ eggs, or the number of resultant larvae (Spearman correlation, *P*>0.05). The egg cohort with 0% rickettsial prevalence (meaning that **not a** **single** egg out of the 10 tested by qPCR contained *Rickettsia* phylotype G021) came from a tick whose eggs exhibited a hatching rate of 94.1% and yielded 1750 viable larvae. Both the hatching rate and the larval count of the cohort were above the mean values of the ciprofloxacin group as a whole (93.2% hatching rate and 1315 viable larvae, respectively). However, the three cohorts with 100% prevalence of rickettsiae had a below-average mean hatching rate of 69.3%, and a mean of 1154 larvae, also lower than the overall mean larval count of the ciprofloxacin group.

**Table 1:** Prevalence of infection with *Rickettsia* species phylotype G021 in *Ixodes pacificus* egg cohorts of 10 from each of the 14 ticks in the ciprofloxacin-injected group.

Number of eggs positive for*Rickettsia* species phylotypeG021 in a cohort	Percentage of cohorts withthe rickettsial prevalence (%)	Averagehatchingrate (%)	Averagenumber oflarvae
0/10	7.1	94.1	1750
2/10	14.3	97.8	836
3/10	7.1	99.2	1640
6/10	7.1	99.0	1272
8/10	28.6	95.0	1513
9/10	14.3	81.6	1093
10/10	21.4	69.3	1154

### Tick oviposition and egg hatching

**Figure 2:**
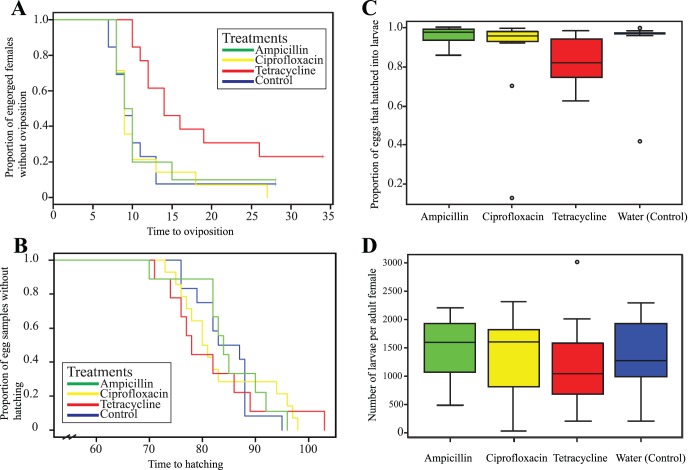
Kaplan-Meier (time-to-event) plots of the preoviposition period and the incubation periods, and boxplots representing egg hatching rate, and the number of larvae from engorged *Ixodes pacificus* females. 2A) A Kaplan-Meier plot where each colored line corresponds to the fraction of adult female ticks that had not started laying eggs on a particular day after the ticks were injected with antibiotics or water (control). Vertical dashes on the colored lines represent censorship, where a tick that had not started to lay eggs died and was dropped from the study. The tetracycline group (red) took significantly longer to start laying eggs compared to the rest (*P*<0.05) 2B) A Kaplan-Meier plot where the colored lines represent the proportion of egg samples laid by individual ticks whose eggs had not finished hatching into larvae a certain number of days after oviposition began. 2C) A boxplot showing the hatching rate, or the fraction of tick eggs that successfully hatched into larvae, in the four treatment groups. Each individually colored box represents the hatching rate distribution of eggs laid by each female tick in the group. 2D) A boxplot specifying the distribution of the total number of larvae each female yielded. No significant difference in (C) hatching rate or (D) the number of larvae was present between treatment groups (*P*<0.05). Circles outside the boxes in (C) and (D) represent outliers. The colors in all the plots in the figure correspond to specific treatment groups: blue - ampicillin, yellow - ciprofloxacin, red - tetracycline, green - injection with water (control).

Ticks in the tetracycline group took a median (95% CI) of 13 (10–16) days after injection treatment to start laying eggs. Ciprofloxacin, water control and ampicillin groups each spent a median of 9 (8–10) days after injection before commencing oviposition, (Figure 2A). Tetracycline treatment significantly delayed *I. pacificus* oviposition compared to all other treatment groups (Log-Rank test, *P*<0.05). The preoviposition periods of the ampicillin, ciprofloxacin, and control groups were not significantly different from each other (Log-Rank test, *P*>0.05).

Several ticks did not lay eggs and were censored in the Kaplan-Meier analysis upon their deaths (Figure 2A). The three ticks that did not lay eggs in the tetracycline group were affected by a pathogen that exhibited a green, mold-like morphology and was presumably fungal. Another tetracycline-treated tick affected by the pathogen laid eggs that never hatched. Two ticks died without laying eggs in the other treatment groups – one in the ampicillin group and one in the control group. These ticks did not have any apparent fungal infection.

The mean (95% CI) duration of incubation averaged 85 (79–91) days in the eggs from ticks injected with water, 84 (81–88) days in ampicillin-injected ticks, 84 (78–89) days in the ciprofloxacin group, and 82 (74–89) days in the tetracycline group (Figure 2B). As expected from the strikingly similar incubation periods, no significant difference was present between any of the treatment groups (ANOVA, *P*>0.05).

Eggs in the ampicillin group had the highest hatching rate of all groups studied, with a mean (95% CI) of 96.8% (94.2%–98.7%) total eggs hatching into larvae (Figure 2C). Ticks in the control group followed, with a mean hatching rate of 94.5% (84.1%–99.5%). The ciprofloxacin group laid eggs with a slightly lower hatching rate of 93.2% (83.6%–98.5%), and only 85.2% (73.8%–94.2%) eggs from tetracycline-injected ticks hatched into larvae. However, the tetracycline group’s lower hatching rate was not significantly different from the hatching rates of other treatment groups (ANOVA, *P>*0.05).

Eggs from the ampicillin group yielded the highest number of larvae of all treatment groups, with a mean (95% CI) number of 1493 (1073–1847) larvae hatched. The control group followed, with an average of 1359 (780–1878) hatched larvae. The ciprofloxacin group’s eggs hatched into 1315 (790–1316) larvae, and the tetracycline group had the lowest mean number of larvae at 1223 (519–1828) (Figure 2D). As with hatching rate, no significant difference was found between any of the groups with respect to the number of larvae (ANOVA, *P*>0.05).

## Discussion

In this study, we used antibiotics to study the effects of an endosymbiotic *Rickettsia* species on reproductive fitness of *I. pacificus*. We have concluded that (1) ciprofloxacin is a considerably more effective anti-rickettsial antibiotic than tetracycline when applied to *I. pacificus* in vivo. (2) Ampicillin does not clear *Rickettsia* species phylotype G021 from engorged females or their eggs, and can therefore be used as an antibiotic control in related studies (3) A substantial decrease in rickettsial density of *I. pacificus* females has no significant effect on the female’s preoviposition period or the number of offspring. Finally, (4) *Rickettsia*-free eggs can successfully hatch into larvae, do not take longer to do so than eggs infected with *Rickettsia* and do not have reduced hatching rates. This suggests that the *Rickettsia* phylotype is unnecessary for successful hatching of *I. pacificus* eggs. In summary, our findings indicate that the phylotype does not appear to have an effect on embryogenesis, oviposition and egg hatching of *I. pacificus.*

### Effects of Antibiotics on *Rickettsia*

Antibiotic injections demonstrated that tetracycline and ciprofloxacin were effective antibacterial agents against *Rickettsia* species phylotype G021 in *I. pacificus* (Figure 1). Quantitative PCR using egg DNA samples showed that ciprofloxacin more effectively decreased rickettsial densities in *I. pacificus* than tetracycline, whereas tetracycline had much greater antibacterial activity on the *Rickettsia* species than ampicillin treatment and the control injection with water (Figure 1D). However, in spent females, rickettsial density in the tetracycline group was not significantly different from ticks injected with ampicillin or water (Figure 1C). The inconsistency between rickettsial densities in adults and eggs most likely resulted from tetracycline’s bacteriostatic properties. Since bacteriostatic agents operate by stopping bacterial growth, qPCR detected *Rickettsia* species that were no longer growing in the tetracycline-injected spent female. On the contrary, bactericidal activity of ciprofloxacin killed the *Rickettsia* species in *I. pacificus*, and we presume that much of the rickettsial DNA from lysed bacteria had been degraded in the ciprofloxacin-treated spent females.

Ticks treated with tetracycline exhibited a significant delay in oviposition compared to ticks in the other treatment groups. The tetracycline group also maintained a consistently lowest average reproductive fitness performance throughout the experiment (Figure 2). In addition, tetracycline-injected ticks were the only ones affected by a lethal mold-like infection, suggesting that the antibiotic treatment may have made *I. pacificus* ticks more susceptible to fungal pathogens. The reason why tetracycline affected oviposition, but ciprofloxacin did not, remains elusive. Antibiotics’ chemical toxicity on arthropods has not been conclusively established, in part because it is often impossible to separate purely chemical, direct toxicity from indirect toxicity that can result from an antibiotic affecting an arthropod’s flora [44]. However, in this study, the tetracycline-induced extension of *I. pacificus*’ preoviposition period was not connected to the antibiotic’s effect on *Rickettsia*, as delayed oviposition was not observed in the ciprofloxacin-injected ticks that had a smaller rickettsial density. This further strengthens our conclusion that ciprofloxacin is a better choice of anti-rickettsial agent to be used on *I. pacificus* in vivo, and potentially a more effective antibiotic to be used on other arthropods in similar studies.

Ampicillin is not effective at killing *Rickettsia* species phylotype G021 or stopping rickettsial growth in *I. pacificus*, consistent with in vitro assays that demonstrated inactivity of beta-lactam antibiotics against rickettsiae [31]. Ampicillin is also ineffective in vitro on other tick-borne intracellular bacteria *Ehrlichia chaffeensis* and *Anaplasma phagocytophilum* [32] and cannot be used to treat patients infected with rickettsial diseases [31]. Interestingly, ampicillin injection into pea aphids (*Acyrthosiphon pisum*) eliminates *Rickettsia* species in a second post-injection generation [25]. Ampicillin-treated ticks and their eggs had slightly lower rickettsial densities than did ticks and eggs in the water control group, but the difference was not statistically significant.

### *Rickettsia*’s effect on embryogenesis, oviposition, and egg hatching of *I. pacificus*

Decreased rickettsial load did not reduce reproductive fitness of engorged *I. pacificus* females and their eggs (Figure 2), or have any other apparent consequence on fecundity, suggesting that the rickettsiae are not required for *I. pacificus* embryogenesis and egg hatching. The tick with the lowest reproductive fitness in the ciprofloxacin group (13% of eggs hatched into a total of 231 larvae) was one of the two ticks in the ciprofloxacin group with 100% *Rickettsia* prevalence in its egg cohort (Table 1). Four out of 14 egg cohorts from ciprofloxacin-injected ticks had rickettsial prevalence of less than 50%, suggesting that less than 50% of all eggs laid by those ticks contained *Rickettsia*. Yet ciprofloxacin-treated ticks’ egg incubation period, egg hatching rate, and the number of larvae were not significantly different from any other group, including the control. These findings strongly suggest that *Rickettsia* phylotype G021 is not necessary for embryogenesis, oviposition, and egg hatching of *I. pacificus.* A large decrease in beneficial symbionts tends to have an **immediate** effect on arthropod fecundity, i.e. a reduced number of progeny and significantly lower hatching rates. This has been demonstrated in *Amblyomma americanum* ticks containing *Coxiella* symbionts [26], as well as in other arthropods. An almost complete elimination of tsetse flies’ *Wigglesworthia* and *Sodalis* symbionts with rifampin- and tetracycline-laden blood led to a decrease in the number of the flies’ offspring and to a decline in the hatching rate from larva into pupa (in tsetse flies, eggs hatch inside the mother, and larvae molt into pupae without feeding; [45]). In a non-blood feeder system, rifampin treatment of secondary symbionts decreases nymphal size in two biotypes of the silverleaf whitefly *Bemisia tabaci* and reduces survival of eggs into adulthood in one of the whitefly’s biotypes [46]. Antibiotic treatment also decreases embryogenesis and egg hatching of a Collembolan *Folsomia candida*, likely because of the antibiotics’ effect on the insect species’ flora [44]. These findings provide a striking contrast with triatomine bugs (Hemiptera: Triatominae), where symbionts are passed on from one life cycle stage to the next by coprophagy. In triatomines, eggs and, therefore, newly emerged larvae do not initially contain the mutualistic symbiont *Rhodococcus rhodnii*; the larvae have to acquire the symbiont by ingesting contaminated feces. Although consumption of sterile feces causes mortality during later larval instars of the triatomine bug, the symbiont is clearly unnecessary for embryogenesis [47].

Our findings do not preclude the possibility that the benefit *Rickettsia* provide to *I. pacificus* may become apparent in later life cycle stages, i.e. larvae, nymphs, and flat (unfed) adults. For example, *Rickettsia* phylotype G021 might be a beneficial symbiont of larval, nymphal, and flat adult stages of *I. pacificus* in the environment by enhancing the host’s general fitness, possibly by protecting the host against stress factors that do not affect the tick in the lab. *Rickettsia*’s likely role in increasing larval motility of ticks *I. scapularis*, *A. americanum,* and *Dermacentor andersonii,* provides an example of a potential fitness benefit [16]. Concerning protection from environmental stress, *Rickettsia* is associated with heat-induced upregulation of structural proteins in the whitefly *B. tabaci* [48].

Future studies can further address the role of *Rickettsia* phylotype G021 in later life cycle stages of *I. pacificus* by creating *Rickettsia*-free tick lines and comparing their reproductive and general fitness to wild type infected ticks. In addition to overall fitness, these experiments can shed more light on the intriguing possibilities for *Rickettsia*’s specific function in the tick, such as increased motility [16]. *Rickettsia* has been found in *I. pacificus*’ relatives, *I. scapularis* [18] and *I. ricinus* [17], but almost nothing is known about those *Rickettsia*-tick relationships. Little is also known about other bacterial residents of *I. pacificus.* A metagenomic study of *I. pacificus* can provide insights into the biology of *I. pacificus* and put the *Rickettsia* symbiont in a broader context.

### Data Availability Statement

A spreadsheet containing the data obtained and analyzed for this study is available for download on Dr. Jianmin Zhong’s laboratory webpage: http://users.humboldt.edu/jzhong/.
